# Intelligence, Correspondence, &c.

**Published:** 1837-01-01

**Authors:** 


### Intelligence, Correspondence, &c.

Our readers are aware that a Public Dinner of Medical Practitioners took place lately in the Borough, attended by more than a Hundred Gentlemen of the Profession, when it was determined to found a "British Medical Association," for promoting harmony in the Profession, and many other useful purposes. Since then, the Provisional Council have been employed in the formation of Rules and Regulations, and getting good legal opinions thereon. They are not yet completed, otherwise they should have been published in our present Number. A General Meeting of Medical Practitioners is to be called, about the 12th of January, in some central part of the Metropolis, and it is to be hoped that a large assemblage will meet on that occasion.—*P.S.* While correcting this proof we received the printed Rules. They will appear in our next.—27*th Dec.* 1836.

Dr. Jameson's Case of Iritis and Blindness from Tic Douloureux in our next.

Dr. Jacob's Communication has been received, and we shall have much pleasure in availing ourselves of it in our next Number.

